# Temporal outcomes and biomarkers of rituximab treatment response in pemphigus: Validation of a historical control dataset

**DOI:** 10.1016/j.xjidi.2026.100510

**Published:** 2026-07-20

**Authors:** Chuda Rujitharanawong, Sasima Tongsai, Aimee S. Payne

**Affiliations:** 1 Department of Dermatology, Columbia University, New York, New York, USA; 2 Department of Dermatology, Faculty of Medicine Siriraj Hospital, Mahidol University, Bangkok, Thailand; 3 Research Department, Faculty of Medicine, Siriraj Hospital, Mahidol University, Bangkok, Thailand

**Keywords:** Autoimmunity, B cells, Blistering disease, Clinical research, Immunotherapy

## Abstract

Rituximab and corticosteroids remain the only clinically approved therapies for pemphigus vulgaris, although novel therapeutics are entering clinical development. A historical control dataset previously described clinical and serologic outcomes after rituximab therapy in pemphigus vulgaris, but whether these data are representative of other patient cohorts is currently unclear. A contemporary pemphigus vulgaris cohort (n = 97) was compared with the historical cohort (n = 107) to assess clinical outcomes as well as desmoglein 3 ELISA predictive performance for achieving complete remission off oral immunosuppressive therapy. A total of 41.2% of patients achieved complete remission off oral immunosuppressive therapy after the first rituximab cycle, with a maximum complete remission off oral immunosuppressive therapy prevalence of 52.8% at month 36, including multiple rituximab cycles. Despite differences in baseline characteristics and treatments (particularly rituximab dosing), the temporal patterns of remission, relapse, and retreatment were broadly similar across cohorts. A desmoglein 3 ELISA index value ≤ 130 RU/ml and > 90.7% titer reduction between months 3 and 9 demonstrated 90% specificity and 88% sensitivity for future achievement of complete remission off oral immunosuppressive therapy, with negative predictive values of 85% and 92%, respectively, suggesting that low and/or rapid large drops in autoantibody titers after rituximab may predict an absence of disease activity. These data describe the temporal outcomes of rituximab therapy in pemphigus vulgaris and validate the historical control dataset for future comparative effectiveness research and biomarker-driven management.

## Introduction

Although rituximab is currently the standard-of-care treatment for pemphigus vulgaris (PV) (Joly et al, 2017; Werth et al, 2021; Murrell et al, 2020), novel therapeutic options for this chronic autoimmune disease continue to evolve. Clinical trials of novel agents in PV that have failed to lead to clinical approval highlight the importance of trial design for clinical outcomes, particularly the choice and timing of primary endpoints (Rujitharanawong et al, 2026). Previously, a historical control dataset based on a single-center cohort was published to establish temporal outcomes after rituximab therapy in PV (Tovanabutra et al, 2022), demonstrating the kinetics of clinical remission and relapse as well as autoantibody titers at defined time points after rituximab infusion—a valuable resource for planning clinical trials in PV. However, whether these results are representative of patients with PV outside a single center is currently unknown.

In parallel, serologic biomarkers, particularly anti-desmoglein 3 (DSG3) antibody titers, have been investigated as predictors of disease activity and treatment response (Harman et al, 2001; Cheng et al, 2002; Abasq et al, 2009; Joly et al, 2017; Albers et al, 2017; Tovanabutra et al, 2022; Hébert et al, 2025). Clinical studies have evaluated the utility of DSG3 autoantibody ELISA thresholds for predicting disease relapse and determining retreatment with rituximab (Abasq et al, 2009; Albers et al, 2017; Genovese et al, 2022; Hébert et al, 2025), whereas other studies have examined the relationship between DSG3 autoantibody titers and the achievement of complete remission after rituximab therapy (Tovanabutra et al, 2022). Notably, independent studies have identified a threshold of > 130 RU/ml for predicting disease relapse (Abasq et al, 2009) and, conversely, ≤ 130 RU/ml for predicting achievement of complete remission off therapy (CROT) (Tovanabutra et al, 2022) after rituximab therapy of pemphigus, highlighting the potential clinical value of this biomarker and need for independent validation in different patient populations. Toward this goal, the historical control dataset was validated through comparison with a contemporary cohort of rituximab-treated patients with PV, focusing on temporal clinical and serologic outcomes.

## Results

### Patient demographics and comparability of historical and contemporary cohorts

A total of 97 patients with PV were included in the analysis. Baseline demographic and clinical characteristics of this contemporary cohort were compared with those of the historical control cohort (n = 107) (Table 1). Cohort comparability was assessed using standardized mean differences (SMDs), where values < 0.1, 0.1–0.2, 0.2–0.5, and > 0.5 indicate negligible, small, moderate, and large differences, respectively. Small differences in median age (SMD = 0.18) and body mass index (SMD = 0.13) were observed between the cohorts. The proportion of male patients (32.0 vs 44.9%; SMD = 0.27) and patients with the mucocutaneous PV subtype (68.0 vs 81.3%; SMD = 0.30) was lower in the current cohort than in the historical cohort, reflecting moderate differences. A large difference (SMD = 1.08) was observed between the cohorts with regard to the initial rituximab treatment regimen; 76 patients (78.4%) in the current cohort received the Food and Drug Administration–approved dosing regimen for PV (1000 mg infusions separated by 2 weeks), whereas 21 patients (21.6%) received the lymphoma-dose regimen (375 mg/m^2^ weekly for 4 weeks), compared with 27.1 and 72.9% in the historical cohort, respectively. With respect to concomitant medications at the time of rituximab initiation, prednisone use was common in both groups, with a moderately higher proportion in the historical cohort (87.9 vs 78.3%; SMD = 0.26). Mycophenolate mofetil use demonstrated large differences between the current and historical cohorts (12.3 vs 43.9%; SMD = 0.70), whereas other steroid-sparing treatments, such as dapsone, doxycycline/tetracycline, azathioprine, intravenous Ig, and plasmapheresis, demonstrated negligible to moderate differences (SMD = 0.02–0.26).

**Table 1:** Baseline Characteristics Were Generally Comparable between the Contemporary and Historical Cohorts, with Small-To-Moderate Differences in Most Demographic, Clinical, and Concomitant Treatment Variables

Characteristic	This Study (n = 97)	Tovanabutra et al (n = 107)	SMDs
Age, y, median (IQR)	50.1 (39.1–62.2)	53.0 (43.5–63.3)	0.18
Sex			0.27
Male	31 (32.0%)	48 (44.9%)	
Female	66 (68.0%)	59 (55.1%)	
Subtypes			0.30
Mucocutaneous pemphigus vulgaris	66 (68.0%)	87 (81.3%)	
Mucosal pemphigus vulgaris	31 (32.0%)	20 (18.7%)	
Body mass index, kg/m^2^, median, (IQR)	27.2 (22.9–30.5)	27.9 (24.6–31.7)	0.13
Rituximab dose regimen, 1^st^ cycle			1.08
1000 mg on days 1 and 15	76 (78.4%)	29 (27.1%)	
375 mg/m^2^ weekly for 4 wk	21 (21.6%)	78 (72.9%)	
Medication at rituximab treatment			
Prednisone	76 (78.3%)	94 (87.9%)	0.25
≤ 10 mg/d	11 (11.3%)	25 (23.4%)	
11–20 mg/d	9 (9.3%)	23 (21.5%)	
21–40 mg/d	20 (20.6%)	23 (21.5%)	
41–60 mg/d	23 (23.7%)	12 (11.2%)	
> 60 mg/d	9 (9.3%)	11 (10.3%)	
Unknown	4 (4.1%)	0 (0.0%)	
Mycophenolate mofetil	12 (12.3%)	47 (43.9%)	0.72
< 1000 mg/d	1 (1.0%)	0 (0.0%)	
1000–1500 mg/d	4 (4.1%)	6 (5.6%)	
2000 mg/d	3 (3.1%)	17 (15.9%)	
2500–3000 mg/d	4 (4.1%)	24 (22.4%)	
Azathioprine	1 (1.0%)	6 (5.6%)	0.26
≤ 1.25 mg/kg/d	1 (1.0%)	1 (0.9%)	
1.26–2.5 mg/kg/d	0 (0.0%)	5 (4.7%)	
Dapsone	4 (4.1%)	5 (4.7%)	0.03
Doxycycline/tetracycline	2 (2.1%)	7 (6.5%)	0.22
Plasmapheresis	0 (0.0%)	3 (2.8%)	0.24
Intravenous Ig	5 (5.2%)	5 (4.7%)	0.02
Other	3 (3.1%)	10 (9.3%)	0.27

Large differences were observed in the initial rituximab regimen and mycophenolate mofetil use between cohorts.

Abbreviations: IQR, interquartile range; SMD, standardized mean difference.

### Temporal trends and rates of CROT after rituximab

Clinical outcomes were evaluated over a 36-month period after the first rituximab cycle, including patients who received additional cycles at the treating physician’s discretion for relapsed or refractory disease. Outcomes were defined according to established consensus criteria (Murrell et al, 2008): CROT was defined as having no new or existing lesions for ≥ 2 months without systemic immunosuppressive therapy other than rituximab, and relapse was defined as having ≥ 3 new lesions per month that did not heal within a week or as extension of existing lesions.

Temporal trends of CROT in the contemporary cohort are illustrated in Figure 1, depicting 4 outcome categories: (i) patients who achieved and maintained CROT after the initial cycle of rituximab (green, denoted as CROT1); (ii) patients with relapsed disease after initial CROT (orange), most of whom received a second cycle of rituximab; (iii) patients who achieved CROT after 2 or more cycles of rituximab (yellow, denoted as CROT2+), either because of the requirement for 2 or more rituximab cycles to achieve first CROT or because of disease relapse and reachievement of CROT after a subsequent rituximab cycle; and (iv) patients who never achieved CROT despite 1 or more rituximab cycles. Among 97 patients, 40 (41.2%) achieved CROT1, with a median time to CROT1 of 6 months (interquartile range = 3.2–8.2). The cumulative CROT rate, including both CROT1 and those who achieved CROT2+, plateaued between months 24 and 36 (Figure 1, green + yellow), with a maximal CROT prevalence of 52.8% at 36 months after the first rituximab infusion. Regarding relapsed and refractory disease (orange + gray, a common enrollment criterion for clinical trials), 47.2–50.0% of patients met these criteria between months 24 and 36, and 35.8–41.4% of patients remained in the nonresponse category during this same time period despite 1 or more cycles of rituximab.

**Figure 1:**
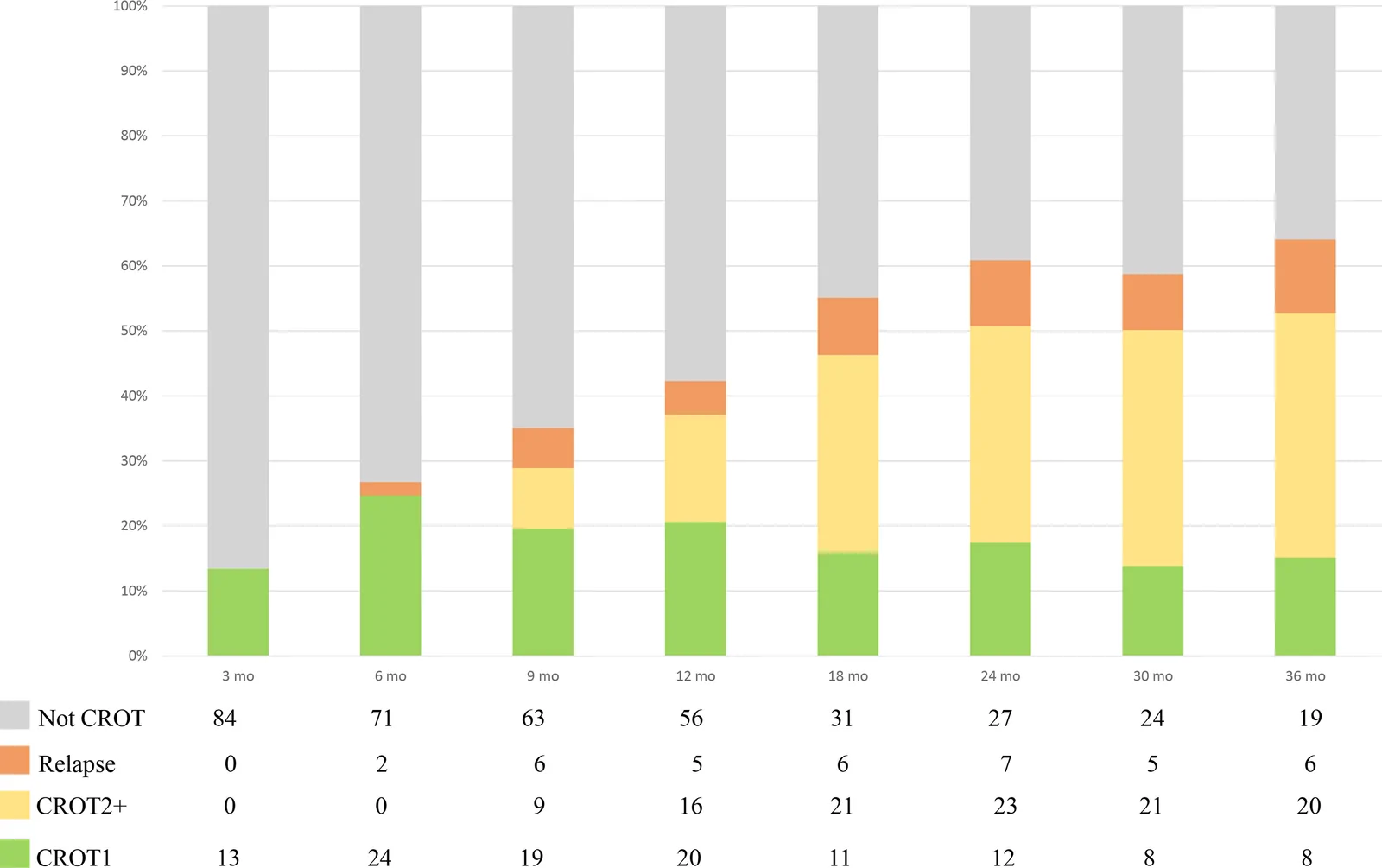
**Time-dependent achievement of CROT after rituximab treatment.** A total of 97 patients with PV were followed for up to 36 months after the first rituximab cycle and censored at the end of follow-up. Graph illustrates the proportion and number of patients achieving and maintaining CROT over time: after the first rituximab cycle (CROT1, green), after 2 or more cycles (CROT2+, yellow), relapse after CROT (orange), and those who did not achieve CROT (gray). CROT, complete remission off therapy; PV, pemphigus vulgaris.

Across historical and contemporary cohorts, remission and relapse outcomes were comparable. The rates of achieving CROT1 were similar (47.7 vs 41.2%, *P* = .39), as were the relapse rates after CROT1 (60.8 vs 65.0%, *P* = .68) (Table 2). Likewise, no significant differences were observed in the proportion of patients achieving CROT2+ (52.4 vs 49.2%, *P* = .72) or in the relapse rates after CROT2+ (51.5 vs 46.7%, *P* = .70). Among patients who achieved CROT1, the median interval between the first and second rituximab infusions was comparable (21.6 vs 18.6 months). In contrast, among non-CROT1 patients, the historical control cohort had a significantly longer interval between the first and second rituximab infusions (12.3 vs 6.5 months).

**Table 2:** Remission, Relapse, and RTX Retreatment Intervals Were Comparable between the Contemporary and Historical Cohorts

Dataset	This Study (n = 97)	Tovanabutra et al (n = 107)	*P*-Value
Rate CROT1	40/97 (41.2%)	51/107 (47.7%)	.356
Rate relapse after CROT1	26/40 (65.0%)	31/51 (60.8%)	.680
Rate CROT2+	30/61 (49.2%)	33/63 (52.4%)	.722
Rate relapse after CROT2+	14/30 (46.7%)	17/33 (51.5%)	.702
Median RTX1–RTX2 interval in CROT1 patients	18.6 (9.5–31.8)	21.6 (13.6–29.2)	.478
Median RTX1–RTX2 interval in non-CROT1 patients	6.5 (5.3–8.2)	12.3 (7.2–19.7)	< .001

Rates of CROT1, CROT2+, and relapse did not differ significantly. The RTX1–RTX2 interval in non-CROT1 patients was significantly shorter than in the contemporary cohort.

Abbreviations: CROT, complete remission off therapy; RTX, rituximab.

Comparing temporal trends by time point across the historical and contemporary cohorts, the proportion of patients achieving CROT1 in the historical cohort was 18.1% at 6 months and peaked at 32.4% at 12 months, whereas in the contemporary cohort, the peak CROT1 response occurred at 6 months (24.7%). Thereafter, rates of CROT1 declined as relapses occurred, and additional rituximab cycles increased the rates of CROT2+, resulting in similar 12-month cumulative CROT rates in the historical and contemporary cohorts (38.1 vs 37.1%, *P* = 1.00). From 24 to 36 months, cumulative CROT rates plateaued in both cohorts. The highest prevalence of CROT was observed at 36 months in both datasets, with rates of 43.1 and 52.8% in the historical and contemporary cohorts, respectively (*P* = .28). Long-term nonresponse rates at 36 months were also similar between the cohorts (36.1 vs 35.8%; *P* = .97).

In both the current and historical cohorts, the lymphoma-dose regimen was associated with a higher proportion of patients achieving CROT1 than was the Food and Drug Administration–approved dosing regimen for PV (59.1 vs 30.7% in the current cohort and 38.5 vs 22.2% in the historical cohort). However, differences between regimens were less pronounced after the second rituximab cycle, with CROT rates of 45.5 versus 48.5% in the current cohort and 42.5 versus 34.8% in the historical cohort, respectively.

### Clinical evaluation of DSG3 ELISA as a biomarker of treatment response

DSG3 autoantibody titers correlate with disease activity when tested within the linear range of the assay (Harman et al, 2001; Cheng et al, 2002). A total of 27 patients with PV were included in the biomarker analysis, contributing 84 longitudinal observations across all time points. There were no statistically significant differences between the CROT and non-CROT groups with regard to age (*P* = .775), sex distribution (*P* = .190), body mass index (*P* = .735), rituximab regimen (*P* > .99), concomitant immunosuppressive therapy (*P* = .183), prednisolone use (*P* = .415), or baseline anti-DSG3 antibody levels (*P* = .852) (Table 3).

**Table 3:** Baseline Characteristics of 27 Patients with PV Included in the Biomarker Analysis, Stratified by CROT Status, with No Significance Between-Group Differences in Demographics, Treatment-Related Variables, or Baseline Anti-DSG3 Levels

Characteristic	Not CROT (n = 8)	CROT (n = 19)	*P*-Value
Age, y	50 ± 17	48 ± 14	.775
Sex, n (%)			
Male	7 (88%)	10 (53%)	.190
Female	1 (13%)	9 (47%)	
BMI, kg/m^2^	26.7 ± 5.5	27.6 ± 6.8	.735
Rituximab regimen, n (%)			> .99
1000 mg (d 1 and 15)	6 (75%)	14 (74%)	
375 mg/m^2^ weekly ×4	2 (25%)	5 (26%)	
Immunosuppressive use, n (%)			.183
No	4 (50%)	4 (21%)	
Yes	4 (50%)	15 (79%)	
Prednisone use, n (%)			.415
No	4 (50%)	6 (32%)	
Yes	4 (50%)	13 (68%)	
Anti-DSG3 level, median (IQR)	120 (96–610)	120 (68–730)	.852

Abbreviations: BMI, body mass index; CROT, complete remission off therapy; DSG3, desmoglein 3; IQR, interquartile range; PV, pemphigus vulgaris.

Longitudinal changes in DSG3 ELISA levels were evaluated using linear mixed-effects models with random intercepts for individual patients. Patients who achieved CROT demonstrated a trend toward a faster initial decline in DSG3 titers than those who did not achieve CROT, both in regard to absolute and percentage change in DSG3 ELISA index value (Figures 2 and 3).

**Figure 2:**
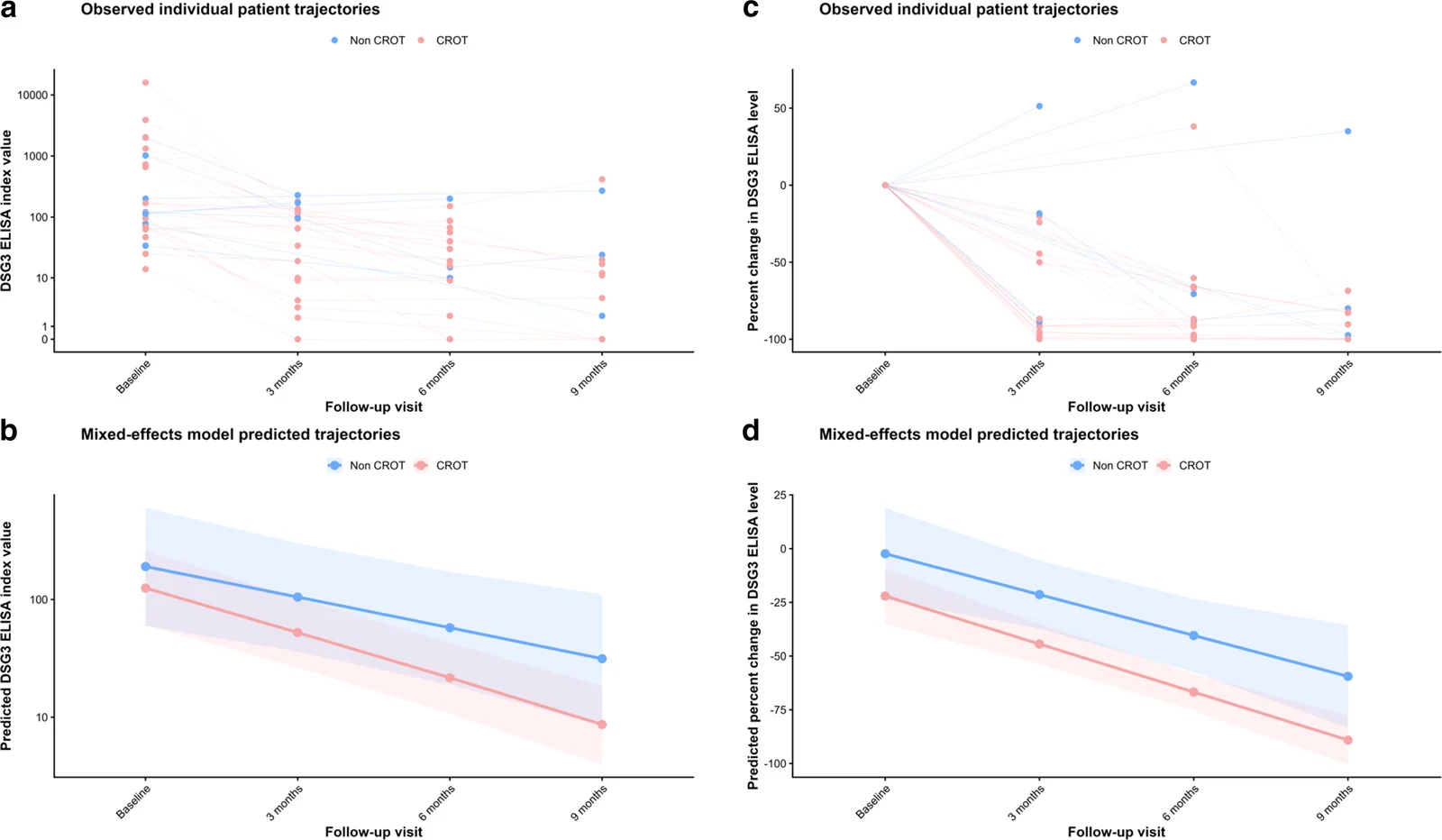
**Longitudinal trajectories of DSG3 ELISA index values according to achievement of CROT (n = 19) or nonachievement of CROT (n = 8).** (**a**) Observed individual patient trajectories from baseline through 9 months of follow-up displayed on a pseudo-logarithmic scale. (**b**) Predicted log-transformed DSG3 ELISA index value trajectories derived from the linear mixed-effects model with corresponding 95% confidence intervals. (**c**) Observed individual patient trajectories from baseline through 9 months of follow-up, displayed as percentage change from baseline. (**d**) Predicted trajectories of percentage change in DSG3 ELISA levels from baseline, derived from the linear mixed-effects model with corresponding 95% confidence intervals. CROT, complete remission off therapy; DSG3, desmoglein 3.

**Figure 3:**
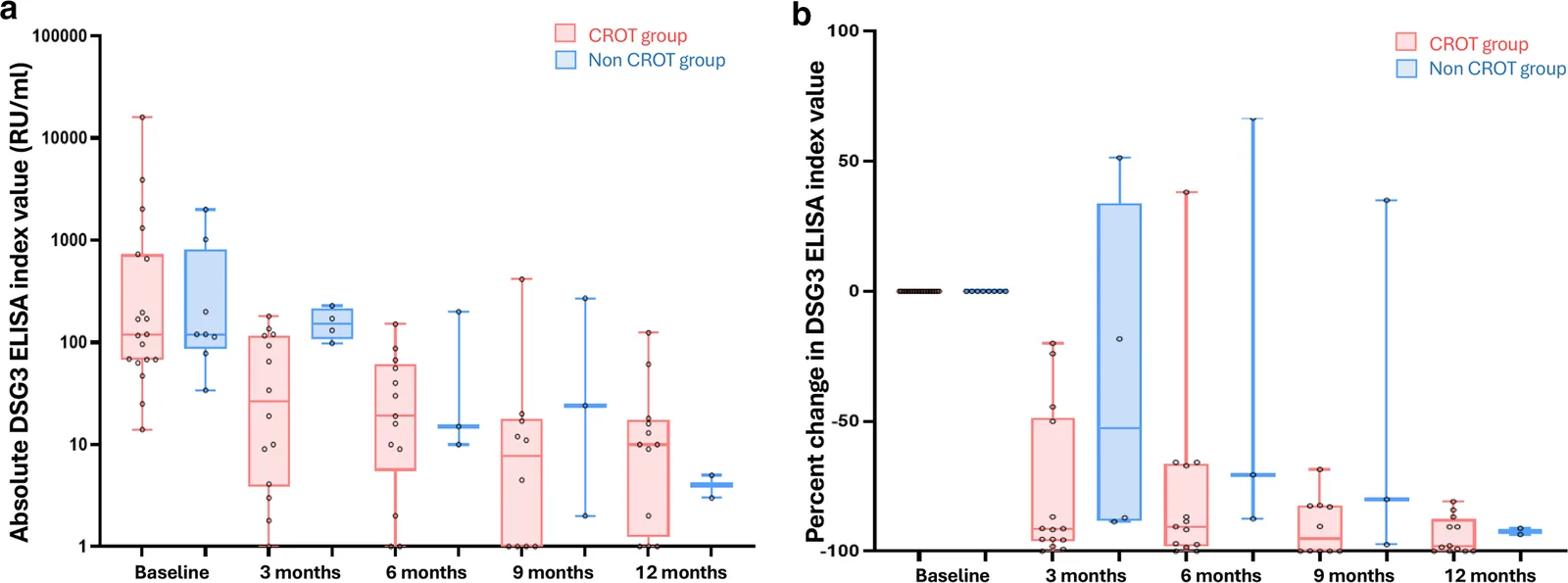
**Temporal changes in desmoglein 3 ELISA index values in patients who did or did not achieve CROT after rituximab treatment.** Measurements are shown at baseline and 3, 6, 9, and 12 months after rituximab. For both panels, box plots display the median and interquartile range, with whiskers representing the minimum and maximum values; individual dots indicate patient-level data. (**a**) Absolute DSG3 ELISA index values (RU/ml). (**b**) Percentage change from baseline in DSG3 ELISA index values. CROT, complete remission off therapy; DSG3, desmoglein 3.

The previously reported historical cohort, using serial dilutions of banked patient samples to assay within the linear range of a commercial DSG3 ELISA (Euroimmun), identified that a > 90.7% reduction in DSG3 autoantibody titers from baseline to months 3–9 was 94.1% sensitive, and an average absolute titer ≤ 130 RU/ml between months 3 and 9 was 95.8% specific for the achievement of future CROT. For the contemporary cohort, DSG3 ELISA index values were derived from laboratory assessments in the electronic medical record, performed by national reference laboratories using a commercial ELISA (Medical & Biological Laboratories), with or without serial dilution. As shown in Table 4, despite these differences, an average DSG3 ELISA index value ≤ 130 RU/ml between months 3 and 9 still demonstrated high specificity (90%), and an average percentage reduction in DSG3 ELISA index value of > 90.7% at months 3–9 demonstrated high sensitivity (88%) for predicting future CROT. Both cutoffs demonstrated strong negative predictive value (NPV): 11 (92%) of 12 patients with a > 90.7% drop in DSG3 ELISA index value from baseline and 17 (85%) of 20 patients with an average DSG3 ELISA index value ≤ 130 RU/ml between months 3 and 9 ultimately demonstrated no disease activity. Similarly, an absolute DSG3 ELISA value < 20 RU/ml between months 6 and 9, which is below the limit of detection for the Euroimmun ELISA but remains low positive on the Medical and Biological Laboratory ELISA depending on the reference laboratory, was associated with a sensitivity of 83%, specificity of 72%, and 93% NPV for achievement of CROT (the latter indicating that 13 of 14 patients with a DSG3 ELISA < 20 RU/ml at any point between months 6 and 9 ultimately demonstrated no disease activity).

**Table 4:** Validation of Historical DSG3 ELISA Cutoff Values for Predicting CROT

	Not CROT (n = 8)	CROT (n = 19)	Sensitivity	Control Study	Specificity	Control Study	PPV	Control Study	NPV	Control Study
Average percentage reduction in DSG3 ELISA index value at mo 3–9										
≤ 90.7%	7	8	88% (53–98)	94% (71–100)	58% (36–77)	67% (45–84)	47% (25–70)	67% (45–84)	92% (65–99)	94% (71–100)
> 90.7%	1	11								
Average DSG3 ELISA index value (RU/ml) between mo 3–9										
> 130	5	2	63% (31–86)	53% (28–77)	90% (69–97)	96% (73–99)	71% (36–92)	90% (55–100)	85% (64–95)	74% (55–88)
≤ 130	3	17								

	Not CROT (n = 6)	CROT (n = 18)	Sensitivity	Control study	Specificity	Control study	PPV	Control study	NPV	Control study
DSG ELISA index value (RU/ml) at any point between mo 6–9										
≥ 20	5	5	83% (44–97)	100% (75–100)	72% (50–88)	57% (34–77)	50% (24–76)	63% (41–80)	93% (69–99)	100% (70–100)
< 20	1	13								

Historical DSG3 ELISA cutoffs demonstrated high sensitivity, specificity, and NPV for predicting future CROT, and absence of disease activity. The 95% CI is shown in parentheses.

Abbreviations: CI, confidence interval; CROT, complete remission off therapy; DSG3, desmoglein 3; NPV, negative predictive value; PPV, positive predictive value.

Longitudinal changes in DSG3 ELISA levels were further evaluated using linear mixed-effects models with random intercepts for individual patients. For longitudinal log-transformed DSG3 ELISA levels, a significant decrease over time was observed (β = −0.197 per month, 95% confidence interval [CI] = −0.324 to −0.070, *P* = .003). Baseline log-transformed DSG3 ELISA levels did not significantly differ between patients who achieved CROT and those who did not (β = −0.416, 95% CI = −1.79 to 0.96, *P* = .544). The interaction between follow-up time and CROT status was not statistically significant (β = −0.089, 95% CI = −0.231 to 0.053, *P* = .216), indicating that longitudinal trajectories of DSG3 ELISA levels were generally similar between groups (Table 4). Similarly, longitudinal percentage changes in DSG3 ELISA levels significantly decreased over time (β = −6.34 per month, 95% CI = −10.0 to −2.65, *P* = .001). However, neither baseline percentage change values (β = −19.7, 95% CI = −44.8 to 5.43, *P* = 0.123) nor the interaction between follow-up time and CROT status (β = −1.11, 95% CI = −5.28 to 3.07, *P* = .599) differed significantly between groups (Table 5).

**Table 5:** Linear Mixed-Effects Models of Longitudinal Log-Transformed DSG3 ELISA Levels and Percentage Changes in DSG3 ELISA Levels According to CROT Status

Variable	β Coefficient	95% CI	*P*-Value
Longitudinal log-transformed DSG3 ELISA levels			
Time months	−0.197	−0.324 to −0.070	.003
CROT	−0.416	−1.79 to 0.96	.544
Time months × CROT	−0.089	−0.231 to 0.053	.216
Longitudinal percentage changes in DSG3 ELISA levels			
Time months	−6.34	−10.0 to −2.65	.001
CROT	−19.7	−44.8 to 5.43	.123
Time months × CROT	−1.11	−5.28 to 3.07	.599

Both outcomes significantly decreased over time, with no significant differences in baseline values or longitudinal trajectories between CROT groups.

Abbreviations: CI, confidence interval; CROT, complete remission off therapy; DSG3, desmoglein 3.

Firth penalized logistic regression analyses were subsequently performed to evaluate the associations between DSG3 ELISA biomarkers and achievement of CROT. Baseline log-transformed DSG3 ELISA levels were not significantly associated with CROT (OR = 1.01, 95% CI = 0.61–1.76, *P* = 1.974). Likewise, although greater 3-month reductions in DSG3 ELISA levels showed a trend toward association with CROT, the relationship did not reach statistical significance (OR = 0.98, 95% CI = 0.94–1.01, *P* = .121) (Tables 6 and 7).

**Table 6:** Multivariable Firth Penalized Logistic Regression Analyses for Predictors of CROT

Variable	Adjusted OR	95% CI	*P*-Value
Baseline log-transformed DSG3 ELISA level (model adjusted for age and sex using baseline data)	1.01	0.61–1.76	.974
3-mo percentage change in DSG3 ELISA level (model adjusted for age and sex using 3-month follow-up data)	0.98	0.94–1.01	.121

Neither baseline log-transformed DSG3 ELISA levels nor 3-month reductions in DSG3 ELISA levels were significantly associated with achievement of CROT.

Abbreviations: CI, confidence interval; CROT, complete remission off therapy; DSG3, desmoglein 3; OR, Odds Ratio.

**Table 7:** Full Multivariable Firth Penalized Logistic Regression Models for Predictors of CROT, Adjusted for Age and Sex, Using Baseline and 3-Month Follow-Up Data

Variable	Adjusted OR	95% CI	*P*-Value
Baseline log-transformed DSG3 ELISA level	1.01	0.61–1.76	.974
Age	1.00	0.95–1.06	.862
Male sex	4.27	0.73–44.84	.111
3-mo percentage change in DSG3 ELISA level	0.98	0.94–1.01	.121
Age	0.97	0.86–1.04	.436
Male sex	0.64	0.02–11.16	.758

Abbreviations: CI, confidence interval; CROT, complete remission off therapy; DSG3, desmoglein 3.

## Discussion

In chronic relapsing diseases such as pemphigus, the cumulative rate of CROT after rituximab therapy may overestimate the prevalence of CROT at any given time point owing to relapses after achieving initial remission, underscoring the value of understanding the temporal outcomes of rituximab therapy at a more granular level. Such knowledge allows for better counseling of patients on what to expect after the initial cycle of rituximab therapy as well as likely clinical outcomes by time point. The data derived from historical control datasets thus provide an important resource to inform the selection and timing of pemphigus clinical trial endpoints and can facilitate future comparative effectiveness research.

A prior study systematically characterized the temporal patterns of clinical and serologic responses after rituximab therapy for PV, which demonstrated the kinetics of remission, relapse, and retreatment during the course of standard-care therapy with rituximab, thereby establishing a historical control dataset for evaluating remission durability and treatment response in pemphigus (Tovanabutra et al, 2022). This study validates this historical control dataset using patient data from a different academic medical center and group of treating physicians. Overall, baseline demographic and clinical characteristics were broadly comparable between cohorts, with small-to-moderate differences observed across most variables. A large difference between the historical and contemporary cohorts was the choice of initial rituximab regimen, with lymphoma-dose administration favored in the historical control cohort (72.9%) and PV dose in the contemporary cohort (78.4%). This difference may in part reflect the timing of the first recorded rituximab treatment at each center; in the historical control cohort, the first recorded rituximab treatment was in 2005, shortly before the landmark case series of rituximab in pemphigus was published using the lymphoma-dose regimen (Joly et al, 2007). In contrast, the first recorded rituximab treatment in the contemporary cohort was in 2010, 4 years after rituximab was clinically approved for rheumatoid arthritis at a dose of 1000 mg administered 2 weeks apart, which increased its adoption as the autoimmune dose regimen. Despite these differences, overall rates of CROT1 were similar between historical (47.7%) and contemporary (41.2%) cohorts, as were remission and relapse rates after the initial and subsequent cycles of rituximab (Figure 1 and Table 2). Notably, the 12-month cumulative CROT rates in the historical and contemporary cohorts (38.1 vs 37.1%) closely mirror the 12-month CROT prevalence of 40.3% in a randomized controlled trial of rituximab in PV (Werth et al, 2021), underscoring the predictive value of the dataset. Although the timing of rituximab retreatment was similar for patients achieving CROT1 (21.6 vs 18.6 months in historical and contemporary cohorts, respectively), the timing of retreatment in patients not achieving CROT1 in the contemporary cohort was significantly shorter than in the historical control cohort (6.5 vs 12.3 months), reflecting greater usage of maintenance or additional dose regimens in patients not achieving CROT in the contemporary cohort.

Longitudinal serologic monitoring can play a role in pemphigus management, because prior analyses have shown that anti-DSG3 levels predict clinical outcomes after rituximab, including the likelihood of relapse (Albers et al, 2017; Genovese et al, 2022; Hébert et al, 2025), as well as the future achievement of CROT (Tovanabutra et al, 2022). In this study, we observed that across longitudinal trajectories, mixed-effects models, and Firth penalized logistic regression analyses (Table 5, Table 6, Table 7), the effect estimates generally pointed in the same direction, with patients who achieved CROT tending to demonstrate greater or more rapid declines in DSG3 ELISA levels. However, the relatively small sample size and limited number of events restricted statistical power and may have increased the risk of overfitting or unstable estimates, thus limiting precision and leading to relatively wide CIs, with none of the between-group comparisons reaching statistical significance. Therefore, the current data do not provide sufficient statistical evidence to establish an association with confidence.

Nevertheless, this study supports the historical control findings in a contemporary cohort, demonstrating that a > 90.7% drop in DSG3 autoantibody titers and an absolute DSG3 ELISA index value ≤ 130 RU/ml averaged across months 3–9 after rituximab infusion are sensitive and specific measures of achieving future CROT, with high NPVs suggesting that low autoantibody titers are correlated with a lack of disease activity in PV (Table 4). The prior historical control dataset also identified DSG3 ELISA seronegativity (< 20 RU/ml measured with the Euroimmun ELISA) between months 6 and 9 as having a 100% NPV for the achievement of CROT, indicating that a negative test result predicts no disease activity. In the contemporary cohort, using a real-world setting based on clinical records from reference laboratories with varying cutoff values for ELISA seronegativity (10–20 RU/ml), a DSG3 ELISA index value < 20 RU/ml still shows 83% sensitivity and a 93% NPV for achieving CROT, supporting the observation that a DSG3 ELISA index value below the limit of detection correlates with a lack of disease activity.

Overall, this study supports the generalizability of rituximab response patterns in PV and provides partial external validation of DSG3 ELISA–based biomarkers. These findings suggest that DSG3 ELISA thresholds and response kinetics are applicable across cohorts, supporting the use of the historical control dataset as a robust reference for clinical trial design and comparative effectiveness research. Larger prospective studies with standardized assays and longitudinal follow-up are warranted to clarify the role of DSG3 autoantibody ELISA dynamics in guiding clinical management.

## Materials and Methods

### Study population

This retrospective cohort study was approved by the Columbia University Institutional Review Board. Pemphigus was diagnosed according to consensus criteria (Murrell et al, 2008). Rituximab was administered at the treating physician’s discretion using a dosing regimen of either 1000 mg on days 1 and 15 or 375 mg/m^2^ weekly for 4 consecutive weeks.

For the historical control cohort (Tovanabutra et al, 2022), eligible subjects were all patients with PV followed at the University of Pennsylvania who were identified through the electronic medical record as having at least 1 year of clinical follow-up after rituximab infusions (encompassing study dates between March 2005 and October 2017). The original dataset (n = 107 patients) was established for temporal outcome analysis and included basic demographic, clinical, laboratory, and treatment variables.

For the contemporary validation cohort, eligible subjects were all patients with PV followed at NewYork-Presbyterian Hospital−affiliated practices who were identified through the electronic medical record as having at least 1 year of clinical follow-up after rituximab infusions (encompassing study dates between January 2010 and October 2025), with complete clinical data to match the variables evaluated in the historical control cohort. Of the 99 eligible subjects, 2 were excluded owing to concomitant immunosuppressive therapy for other conditions, leaving a total of 97 cases.

### Validation framework

Data were captured from electronic health records and verified against the data collection criteria of the historical control dataset to ensure consistency in coding and variable definitions. The historical data were sourced from previously published studies and a systematically maintained institutional database, with inclusion and exclusion criteria that were robustly applied and consistent with those of the contemporary cohort, thereby minimizing selection bias and strengthening the validity of cross-cohort comparisons. To assess the representativeness of the historical control dataset relative to the contemporary cohort, key demographic, clinical, and treatment variables were compared using SMDs.

### Outcome measurements

Clinical outcomes were defined according to established consensus criteria (Murrell et al, 2008). The primary endpoint was CROT, defined as the absence of new or established lesions for ≥ 2 consecutive months without systemic immunosuppressive therapy, with no active cutaneous or mucosal disease. Relapse was defined as the occurrence of ≥ 3 new lesions per month, the extension of existing lesions after disease control, or the need to reinitiate or escalate systemic therapy. These definitions were applied consistently across all time points.

### Statistical analysis

All statistical analyses were conducted using R software (version 4.5.3). Baseline characteristics were analyzed at the patient level, whereas longitudinal analyses incorporated repeated measurements within patients. Categorical variables were summarized as frequencies and percentages and compared using the Pearson chi-square test or Fisher’s exact test, as appropriate. Continuous variables were summarized as mean ± SD for normally distributed data or median with interquartile range for non-normally distributed data. For comparability with previously published studies, medians and interquartile ranges were converted to approximate means and SDs using established methods described by Wan et al (2014) and Luo et al (2018), where necessary. Group comparisons for continuous variables were performed using the 2-sample *t*-test for normally distributed data or the Mann–Whitney *U* test for non-normally distributed data, as appropriate. All statistical tests were 2-sided, and a *P* < .05 was considered statistically significant. To compare baseline characteristics between the present cohort and the historical cohort reported by Tovanabutra et al (2022), SMDs were calculated to quantify the magnitude of differences independent of sample size. For continuous variables, SMDs were computed as the difference in means divided by the pooled SD, following the approach described by Andrade (2020) and Cohen et al (1988). Because the historical study reported continuous variables as median and interquartile range, the mean and SD were approximated using the method proposed by Wan et al (2014). For categorical variables, SMDs were calculated on the basis of differences in proportions using the method described by Austin et al (2009). For variables originally reported in multiple categories, categories were collapsed into binary variables (eg, use vs nonuse) to facilitate comparability and SMD estimation. An SMD < 0.1 was considered indicative of negligible imbalance, with values of 0.1–0.2, 0.2–0.5, and > 0.5 representing small, moderate, and large differences, respectively. Comparisons between the contemporary and historical cohorts were interpreted descriptively, and no formal hypothesis testing was performed for these cross-cohort comparisons, in accordance with recommendations for observational comparative studies.

### DSG3 ELISA biomarker analysis

Serum anti-DSG3 antibody levels were extracted from clinical chart records on the basis of assessments performed by nationwide commercial reference laboratories. A negative test result was defined as a > 90.7% average percentage reduction in DSG3 ELISA index value at months 3–9, an average DSG3 ELISA index value ≤ 130 RU/ml between months 3 and 9, or a DSG3 ELISA index value < 20 RU/ml at any point between months 6 and 9. The diseased condition (“Not CROT”) and healthy condition (“CROT”) were used to calculate the sensitivity, specificity, positive predictive value, and NPV of previously identified ELISA cutoff values (Tovanabutra et al, 2022).

Longitudinal trajectories of both log-transformed DSG3 ELISA levels and percentage changes in DSG3 ELISA levels were evaluated using linear mixed-effects models with patient-specific random intercepts fitted using the lme4 package. Fixed effects included follow-up time, CROT status, and the interaction between follow-up time and CROT status. Follow-up time was modeled as a continuous variable in months. Longitudinal trajectory figures were truncated at 9 months for visualization purposes because of substantial missing data at the 12-month follow-up visit.

To evaluate the associations between DSG3 ELISA biomarkers and the achievement of CROT, multivariable Firth penalized logistic regression models were fitted using the logistf package to reduce small-sample bias and potential separation issues. Baseline log-transformed DSG3 ELISA levels and 3-month percentage changes in DSG3 ELISA levels were analyzed separately as predictors of CROT. Models were adjusted for age and sex.

## Ethics Statement

This study was approved by the Institutional Review Board of Columbia University Irving Medical Center. Written informed consent was not required for this retrospective cohort study, which was performed by chart review with a Health Insurance Portability and Accountability Act waiver.

## Data availability statement

The data that support the findings of this study are available from the corresponding author, ASP, upon reasonable request.

## ORCIDs

Aimee S. Payne: http://orcid.org/0000-0001-9389-7918

Chuda Rujitharanawong: http://orcid.org/0000-0003-2164-1678

Sasima Tongsai: http://orcid.org/0000-0002-2310-8888

## Conflict of Interest

ASP is cofounder and cochair of the Scientific Advisory Board of Cabaletta Bio (equity, research support, patent licensing) and serves as a consultant for Janssen and GlycoEra. The remaining authors state no conflict of interest.

## References

[bib1] Abasq C., Mouquet H., Gilbert D., Tron F., Grassi V., Musette P., Joly P. (2009). ELISA testing of Anti-desmoglein 1 and 3 antibodies in the management of pemphigus. Arch Dermatol.

[bib2] Albers L.N., Liu Y., Bo N., Swerlick R.A., Feldman R.J. (2017). Developing biomarkers for predicting clinical relapse in pemphigus patients treated with rituximab. J Am Acad Dermatol.

[bib3] Austin P.C. (2009). Balance diagnostics for comparing the distribution of baseline covariates between treatment groups in propensity-score matched samples. Statistics in Medicine.

[bib4] Andrade C. (2020). Mean Difference, Standardized Mean Difference (SMD), and Their Use in Meta- Analysis: As Simple as It Gets. J Clin Psychiatry.

[bib5] Cheng S.W., Kobayashi M., Kinoshita-Kuroda K., Tanikawa A., Amagai M., Nishikawa T. (2002). Monitoring disease activity in pemphigus with enzyme-linked immunosorbent assay using recombinant desmogleins 1 and 3. Br J Dermatol.

[bib6] Cohen J. (1988).

[bib7] Genovese G., Maronese C.A., Casazza G., Corti L., Venegoni L., Muratori S. (2022). Clinical and serological predictors of relapse in pemphigus: a study of 143 patients. Clin Exp Dermatol.

[bib8] Harman K.E., Seed P.T., Gratian M.J., Bhogal B.S., Challacombe S.J., Black M.M. (2001). The severity of Cutaneous and oral pemphigus are related to desmoglein 1 and 3 antibody levels. Br J Dermatol.

[bib9] Hébert V., Hamwi S., Tancrède-Bohin E., Quéreux G., Pham-Ledard A., Caux F. (2025). Optimizing Pemphigus Management With Rituximab and Short-Term Relapse Predictors. JAMA Dermatol.

[bib10] Joly P., Mouquet H., Roujeau J.C., D'Incan M., Gilbert D., Jacquot S. (2007). A single cycle of rituximab for the treatment of severe pemphigus. N Engl J Med.

[bib11] Joly P., Maho-Vaillant M., Prost-Squarcioni C., Hebert V., Houivet E., Calbo S. (2017). First-line rituximab combined with short-term prednisone versus prednisone alone for the Treatment of pemphigus (Ritux 3): a prospective, multicenter, parallel-group, open-label randomised trial. Lancet.

[bib12] Luo D., Wan X., Liu J., Tong T. (2018). Optimally estimating the sample mean from the sample size, median, mid-range, and/or mid-quartile range. Stat Methods Med Res.

[bib13] Murrell D.F., Dick S., Ahmed A.R., Amagai M., Barnadas M.A., Borradori L. (2008). Consensus Statement on definitions of disease, end points, and therapeutic response for pemphigus. J Am Acad Dermatol.

[bib14] Murrell D.F., Peña S., Joly P., Marinovic B., Hashimoto T., Diaz L.A. (2020). Diagnosis and management of pemphigus: Recommendations of an international panel of experts. J Am Acad Dermatol.

[bib15] Rujitharanawong C., Werth V.P., Payne A.S. (2026). Boom or Bust: Clinical Trials in Pemphigus and Other B-Cell-Mediated Autoimmune Diseases. J Invest Dermatol.

[bib16] Tovanabutra N., Bax C.E., Feng R., Kushner C.J., Payne A.S. (2022). Temporal Outcomes after Rituximab Therapy for Pemphigus Vulgaris. J Invest Dermatol.

[bib17] Wan X., Wang W., Liu J., Tong T. (2014). Estimating the sample mean and standard deviation from the sample size, median, range and/or interquartile range. BMC Medical Research Methodology.

[bib18] Werth V.P., Joly P., Mimouni D., Maverakis E., Caux F., Lehane P. (2021). Rituximab versus Mycophenolate Mofetil in Patients with Pemphigus Vulgaris. N Engl J Med.

